# Supplementary material for: Protection of metal interfaces against hydrogen-assisted cracking
Source: Nat Commun. 2025 Dec 10;16:11032. doi: 10.1038/s41467-025-67310-6 (PMC12696016; doi:10.1038/s41467-025-67310-6)
Supplement: Supplementary file 1 — Supplementary Information [file 41467_2025_67310_MOESM1_ESM.pdf]

Supplementary information of "Protection of metal interfaces against hydrogen-assisted cracking"

Guillaume Hachet[1,2*], Shaolou Wei[1], Ali Tehranchi[1,3], Xizhen Dong[1], Jeremy Lestang[2], Aochen Zhang[4], Binhan Sun[4,5*], Stefan Zaefferer[1], Baptiste Gault[1,2], Dirk Ponge[1*], Dierk Raabe[1]

[1]Max Planck Institute for Sustainable Materials, Düsseldorf, 40237, Germany.
[2]Univ Rouen Normandie, INSA Rouen Normandie, CNRS, Normandie Univ, GPM UMR 6634, Rouen, 76000, France.
[3]Federal Institute for Materials Research and Testing, Berlin, 12489, Germany.
[4]Key Laboratory of Pressure Systems and Safety, Ministry of Education, School of Mechanical and Power Engineering, East China University of Science and Technology, Shanghai, 200237, China.
[5]State Key Laboratory of Chemical Safety, East China University of Science and Technology, East China University of Science and Technology, Shanghai, 200237, China.

*Corresponding author(s). E-mail(s): guillaume.hachet@cnrs.fr; binhan.sun@ecust.edu.cn; d.ponge@mpie.de;

1 Supplementary Note 1: Theoretical analysis determining the most suited solute against hydrogen segregation at crystalline defects

First, the hydrogen susceptibility of different steel grades is presented in Fig. 1.a of the manuscript. They are plotted from data provided in supplementary tables 1, 2, 3, and 4 for martensitic, medium Mn, dual phase, and duplex steels, respectively. These systems were chosen because they represented a wide range of steel microstructures used in contact with hydrogen.

Supplementary Table 1 Details of the data presented in Fig.1.a of the manuscript for martensitic steels. UTS is the ultimate tensile strength, HEI is the hydrogen embrittlement index (HEI), and C_H is the hydrogen concentration.

Reference	Chemical composition (wt%)	UTS (MPa)	HEI (%)	C_H (wppm)	H charging conditions	Strain rate (s^{-1})
[1]	Fe-$C_{0.23}$ -$Mn_{1.21}$-$B_{0.0024}$	1700	70		H_2SO_4 0.5M + NH_4SCN 0.25 g/L i = -0.5 mA/cm^2, t = 24h	9.5×10^{-5}
[2]	Fe-$C_{0.23}$ -$Mn_{1.2}$-$B_{0.002}$	1600	96	3.2	NaCl 3. wt.% + NH_4SCN 3 g/L i = -5 mA/cm^2, t = 24h	2.4×10^{-5}
[3]	Fe-$C_{0.2}$	1500	90	4.2	NaCl 3. wt.% + NH_4SCN 3 g/L i = -1 mA/cm^2, t = 24h	8.3×10^{-5}
[4]	Fe-$C_{0.2}$-$Mn_{3.02}$	1500	94	0.9	NaCl 3. wt.% + NH_4SCN 3 g/L i = -0.1 mA/cm^2, t = 24h	7.5×10^{-5}
[5]	Fe-$C_{0.32}$ -$Mn_{1.15}$-$B_{0.0025}$	1950	85	0.22	NaOH 0.1 M i = -3 mA/cm^2, t = 24h	1.0×10^{-4}
[6]	Fe-$C_{0.1}$-$Mn_{0.02}$	1050	96	10.8	H_2SO_4 0.5M + As_2O_3 0.2 mg/L i = -10 mA/cm^2, t = 24h	8.3×10^{-6}
[6]	Fe-$C_{0.4}$-$Mn_{0.02}$	1300	97	13.7	H_2SO_4 0.5M + As_2O_3 0.2 mg/L i = -10 mA/cm^2, t = 24h	8.3×10^{-6}
[6]	Fe-$C_{0.2}$-$Mn_{0.02}$	1550	91	4.1	NaCl 3. wt.% NH_4SCN 3 g/L i = -0.1 mA/cm^2, t = 24h	8.3×10^{-6}
[7]	Fe-$C_{0.2}$-$Mn_{1.50}$	1400	90	2.3	H_2SO_4 0.1M + NH_4SCN 3 g/L i = -1.25 mA/cm^2, t = 15h	7.5×10^{-5}

Supplementary Table 2 Details of the data presented in Fig.1.a of the manuscript for medium manganese steels.

Reference	Chemical composition (wt%)	UTS (MPa)	HEI (%)	C_H (wppm)	H charging conditions	Strain rate (s^{-1})
[8]	Fe-C$_{0.2}$-Mn$_7$	1400<br>1200	74<br>88	1.2<br>1.2	NaCl 3. wt.% + NH_4SCN 3 g/L<br>i = -5 mA/cm^2, t = 3h	1×10^{-4}
[9]	Fe-C$_{0.09}$-Mn$_{10}$	950<br>1050	81<br>58	6.32<br>6.4	NaCl 3. wt.% + NH_4SCN 3 g/L<br>i = -5 mA/cm^2, t = 24h	1×10^{-5}
[10]	Fe-C$_{0.05}$-Mn$_{12}$	810<br>810	89<br>59	3.07<br>2.42	NaCl 3. wt.% + NH_4SCN 3 g/L<br>i = -5 mA/cm^2, t = 24h	1×10^{-5}
[11]	Fe-C$_{0.05}$-Mn$_{12}$	1330<br>1210	44<br>4	0.5<br>0.4	NaCl 3. wt.% + NH_4SCN 3 g/L<br>i = -5 mA/cm^2, t = 1h	1×10^{-5}
[11]	Fe-C$_{0.05}$-Mn$_{12}$	1330<br>1210	66<br>11	1.5<br>0.9	NaCl 3. wt.% + NH_4SCN 3 g/L<br>i = -5 mA/cm^2, t = 2h	1×10^{-5}
[11]	Fe-C$_{0.05}$-Mn$_{12}$	1330<br>1210	82<br>13	2.0<br>1.6	NaCl 3. wt.% + NH_4SCN 3 g/L<br>i = -5 mA/cm^2, t = 3h	1×10^{-5}
[11]	Fe-C$_{0.05}$-Mn$_{12}$	1330<br>1210	94<br>50	3.4<br>2.6	NaCl 3. wt.% + NH_4SCN 3 g/L<br>i = -5 mA/cm^2, t = 6h	1×10^{-5}
[11]	Fe-C$_{0.05}$-Mn$_{12}$	1330<br>1210	97<br>88	4.1<br>3.7	NaCl 3. wt.% + NH_4SCN 3 g/L<br>i = -5 mA/cm^2, t = 9h	1×10^{-5}
[11]	Fe-C$_{0.05}$-Mn$_{12}$	1330<br>1210	98<br>97	4.4<br>4.2	NaCl 3. wt.% + NH_4SCN 3 g/L<br>i = -5 mA/cm^2, t = 12h	1×10^{-5}

Supplementary Table 3 Details of the data presented in Fig.1.a of the manuscript for dual phase steels.

Reference	Chemical composition (wt%)	UTS (MPa)	HEI (%)	C_H (wppm)	H charging conditions	Strain rate (s^{-1})
[12]	Fe-C$_{0.17}$-Mn$_{1.96}$	1375	24		H_2SO_4 0.5 M + NH_4SCN 3 g/L<br>i = -0.04 mA/cm^2	1×10^{-5}
[12]	Fe-C$_{0.17}$-Mn$_{1.96}$	1375	55		H_2SO_4 0.5 M + NH_4SCN 3 g/L<br>i = -0.06 mA/cm^2	1×10^{-5}
[13]	Fe-C$_{0.08}$-Mn$_{0.5}$	1100<br>1150	38<br>57		NaOH 0.1 M<br>V = -1.6 V, t=24h	3.5×10^{-6}
[13]	Fe-C$_{0.08}$-Mn$_{0.5}$	850<br>900	43<br>50		NaOH 0.1 M<br>V = -1.6 V, t=24h	3.5×10^{-6}
[14]	Fe-C$_{0.15}$-Mn$_{2.5}$<br>Fe-C$_{0.18}$-Mn$_{2.5}$<br>Fe-C$_{0.20}$-Mn$_{2.5}$	659<br>826<br>1097	56<br>59<br>80	2.07<br>2.2<br>2.67	H_2SO_4 0.5 M + CH_4N_2S 1 g/L<br>i = -1 mA/cm^2, t = 2 h	1×10^{-4}
[15]	Fe-C$_{0.21}$-Mn$_{1.3}$	800<br>1050<br>1140<br>1300	20<br>38<br>43<br>68		H_2SO_4 0.5 M + $Na_4P_2O_7$ 1 g/L<br>i = -10 mA/cm^2, t = 2 h	1×10^{-4}

These data show that martensitic steel is the most susceptible to hydrogen embrittlement compared to Medium Mn, dual-phase, and duplex steels. For martensitic steels, different strategies have been developed to reduce HE, like adding elements to form carbides to trap hydrogen [1, 2, 5], or even reducing the grain size of the microstructure [1].

Then, the theoretical influence of doping solute on the interaction between hydrogen and grain boundaries (GBs) using *ab initio* calculations is presented in Fig. 1.c of the manuscript. The literature has already investigated this issue, and we performed complementary *ab initio* calculations for B, C, and N on $\Sigma5(210)$ α-Fe GB. All values are presented in the supplementary table 5.

Supplementary Table 4 Details of the data presented in Fig.1.a of the manuscript for duplex steels.

Reference	Chemical composition (wt%)	UTS (MPa)	HEI (%)	C_H (wppm)	H charging conditions	Strain rate (s^{-1})
[16]	Fe-$C_{0.025}$-$Mn_{1.43}$ -$Ni_{5.64}$-$Cr_{22.8}$	885	82.5	11.4	Molten salts bath T = 200°C, V = -1.5 V	1×10^{-6}
[17]	Fe-$C_{0.025}$-$Mn_{1.37}$ -$Ni_{5.42}$-$Cr_{22.7}$	787<br>797	47<br>57		H_2SO_4 1 M + NaCl 3. wt.% + As_2O_3 250 mg/L i = -0.1 mA/cm^2, t = 24 h	6×10^{-6}
[17]	Fe-$C_{0.025}$-$Mn_{1.37}$ -$Ni_{5.42}$-$Cr_{22.7}$	787<br>797	49<br>50	27<br>21	H_2SO_4 1 M + NaCl 3. wt.% + As_2O_3 250 mg/L i = -0.5 mA/cm^2, t = 24 h	6×10^{-6}
[18]	Fe-$C_{0.026}$-$Mn_{1.57}$ -$Ni_{5.49}$-$Cr_{22.6}$	947	37		H_2SO_4 0.05 M + KSCN 1 g/L i = -1 mA/cm^2, t = 24 h	4×10^{-4}
[18]	Fe-$C_{0.026}$-$Mn_{1.57}$ -$Ni_{5.49}$-$Cr_{22.6}$	947	31		H_2SO_4 0.05 M + KSCN 1 g/L i = -5 mA/cm^2, t = 24 h	4×10^{-4}
[19]	Fe-$C_{0.026}$-$Mn_{1.57}$ -$Ni_{5.43}$-$Cr_{22.9}$	680	25	45	NaOH 0.1 M i = -20 mA/cm^2, *in situ*	2.6×10^{-7}
[19]	Fe-$C_{0.026}$-$Mn_{1.57}$ -$Ni_{5.43}$-$Cr_{22.9}$	680	19	36	H_2SO_4 0.1 M i = -20 mA/cm^2, *in situ*	2.6×10^{-7}

Moreover, the influence of solute X on hydrogen segregation into GB, available in the literature, is presented differently depending on the work. Therefore, these energies are adapted to be compared with our calculations further. In the work of Song *et al.* [20], the solution energy of H in a solute-doped grain boundary ($E^{\mathrm{Sol}}_{\mathrm{H-X-GB}}$) is shown and defined as:

$$E^{\mathrm{Sol}}_{\mathrm{H-X-GB}} = (E^{\mathrm{SC}}_{\mathrm{H-X-GB}} - E^{\mathrm{SC}}_{\mathrm{X-GB}}) - \frac{1}{2}E_{\mathrm{H_2}} \quad (1)$$

with $E^{\mathrm{SC}}_{\mathrm{H-X-GB}}$ and $E^{\mathrm{SC}}_{\mathrm{X-GB}}$, the energies of the supercell containing the GB and the solute X with and without hydrogen, respectively. The energy E_{H_2} is the energy of an isolated H_2 molecule. Consequently, $E^{\mathrm{inter}}_{\mathrm{H-X-GB}}$ is determined by adding a factor $\Delta_{\mathrm{Sol-Inter}}$ to $E^{\mathrm{Sol}}_{\mathrm{H-X-GB}}$, which is:

$$\Delta_{\mathrm{Sol-Inter}} = \frac{1}{2}E_{\mathrm{H_2}} - (E^{\mathrm{SC}}_{\mathrm{H-bulk}} - E^{\mathrm{SC}}_{\mathrm{bulk}}) \quad (2)$$

with $E^{\mathrm{SC}}_{\mathrm{H-bulk}}$ and $E^{\mathrm{SC}}_{\mathrm{bulk}}$ the energies of the bulk supercell with and without hydrogen introduced in a tetrahedral site, respectively. Using parameters from our calculations, $\frac{1}{2}E_{\mathrm{H_2}}$ is -3.38 eV and ($E^{\mathrm{SC}}_{\mathrm{H-bulk}} - E^{\mathrm{SC}}_{\mathrm{bulk}}$) is -3.21 eV, resulting to $\Delta_{\mathrm{Sol-Inter}} = $ -0.17 eV. Consequently, this factor is added for all values of Song *et al.* [20] to present $E^{\mathrm{inter}}_{\mathrm{H-X-GB}}$. The interaction energy provided in Kulkov *et al.* [22] is also a solution energy, so the factor $\Delta_{\mathrm{Sol-Inter}}$ has also been added for their energies.

In the work of Kholtobina *et al.* [21], the binding energy between hydrogen and a solute atom at the GB, $E^{\mathrm{bind}}_{\mathrm{H-X-GB}}$, is defined as:

$$E^{\mathrm{bind}}_{\mathrm{H-X-GB}} = \frac{E^{\mathrm{inter}}_{\mathrm{H-GB}} + E^{\mathrm{inter}}_{\mathrm{X-GB}}}{2} - E^{\mathrm{coseg}}_{\mathrm{H-X-GB}}, \quad (3)$$

with $E^{\mathrm{coseg}}_{\mathrm{H-X-GB}}$ the co-segregation of hydrogen and a second solute into a GB, defined as:

$$E^{\mathrm{coseg}}_{\mathrm{H-X-GB}} = E^{\mathrm{SC}}_{\mathrm{H-X-GB}} - E^{\mathrm{SC}}_{\mathrm{GB}} - (E^{\mathrm{SC}}_{\mathrm{H-bulk}} - E^{\mathrm{SC}}_{\mathrm{bulk}}) - (E^{\mathrm{SC}}_{\mathrm{X-bulk}} - E^{\mathrm{SC}}_{\mathrm{bulk}}). \quad (4)$$

with $E^{\mathrm{SC}}_{\mathrm{GB}}$ is the energy of the supercell with a GB. Therefore, the interaction between hydrogen and a solute-doped GB ($E^{\mathrm{inter}}_{\mathrm{H-X-GB}}$), becomes:

$$E^{\mathrm{inter}}_{\mathrm{H-X-GB}} = E^{\mathrm{coseg}}_{\mathrm{H-X-GB}} - E^{\mathrm{inter}}_{\mathrm{X-GB}}. \quad (5)$$

Here, $E^{\mathrm{coseg}}_{\mathrm{H-X-GB}}$ is determined using eq. (4) with the data presented in Kholtobina *et al.* [21].

In the work of Schuler *et al.* [24], the cosegregation of hydrogen and phosphorous in GB is presented as a binding energy ($E^{\mathrm{inter}} = -E^{\mathrm{bind}}$). Therefore, the energy $E^{\mathrm{inter}}_{\mathrm{H-X-GB}}$ is determined

Supplementary Table 5 Energies (E^{inter}_{H-X-GB} and E^{coh}_{X-GB}) plotted in Fig. 1.a of the manuscript (eV).

X	Reference	E^{inter}_{H-X-GB} (eV)	E^{coh}_{X-GB} (eV)
B	Present work	-0.14	-1.03
B	Present work	0.22	-1.03
B	[20]	0.38	-0.86
B	[20]	-0.46	-0.86
B	[21]	0.72	-0.73
B	[22]	0.57	-0.76
B	[22]	0.57	-0.73
C	Present work	0.10	-0.49
C	[20]	0.07	-0.51
C	[20]	-0.45	-0.51
C	[21]	0.20	-0.46
N	Present work	0.189	-0.098
N	Present work	-0.167	-0.098
N	[21]	0.17	0.13
N	[23]	0.037	0.24
Al	[20]	-0.32	-0.08
Al	[20]	-0.42	-0.08
Al	[21]	0.06	0.00
Si	[20]	-0.07	-0.14
Si	[20]	-0.41	-0.14
Si	[21]	0.12	-0.08
P	[20]	-0.02	0.30
P	[20]	-0.40	0.30
P	[24]	-0.64	1.79
P	[24]	-0.61	1.79
P	[24]	-0.40	1.77
P	[24]	-0.06	1.89
P	[24]	-0.32	1.86
P	[24]	-0.60	1.81
S	[20]	-0.05	1.28
S	[20]	-0.39	1.28
Ti	[20]	-0.46	-0.07
Ti	[20]	-0.41	-0.07
Ti	[21]	0.05	-0.12
V	[20]	-0.24	-0.21
V	[20]	-0.27	-0.21
V	[21]	-0.13	-0.25
V	[25]	-0.47	-0.27
V	[25]	-0.45	-0.27
Cr	[20]	-0.25	-0.10
Cr	[20]	-0.22	-0.10
Cr	[21]	-0.18	-0.16
Cr	[25]	-0.56	-0.23
Cr	[25]	-0.56	-0.23
Mn	[20]	-0.24	-0.49
Mn	[20]	-0.18	-0.49
Mn	[21]	0.02	-0.03
Mn	[25]	-0.51	-0.30
Mn	[25]	-0.55	-0.30
Zr	[21]	0.36	0.37
Co	[20]	-0.26	-0.16
Co	[20]	-0.18	-0.16
Ni	[20]	-0.24	0.07
Ni	[20]	-0.18	0.07
Cu	[20]	-0.26	0.43
Cu	[20]	-0.23	0.43
Nb	[20]	-0.26	-0.34
Nb	[20]	-0.25	-0.34
Nb	[21]	0.36	-0.57
Mo	[20]	-0.25	-0.56
Mo	[20]	-0.21	-0.56
Mo	[21]	0.03	-0.59
Ta	[21]	0.16	-0.7
W	[20]	-0.24	-1.01
W	[20]	-0.16	-1.01
W	[21]	0.02	-0.98
Re	[21]	-0.25	-0.56

using:

$$E^{\text{inter}}_{\text{H}-\text{X}-\text{GB}} = -(E^{\text{coseg,b}}_{\text{H}-\text{X}-\text{GB}} - E^{\text{bind}}_{\text{X}-\text{GB}}). \tag{6}$$

Finally, the segregation energies of hydrogen (and carbon) in Subramanyam *et al.* [25] are defined similarly as the interaction energies of hydrogen for our work. So, they are presented without any modifications.

The interaction of a doping solute with iron, carbon (at high concentration in GB for steels), and GB has been investigated to design crystalline defects resistant to HE. The interaction between X, iron, and carbon is quantified through the mixing enthalpy of a solute X with iron, $H^{\text{mix}}_{\text{X}-\text{Fe}}$, and with carbon, $H^{\text{mix}}_{\text{X}-\text{C}}$, presented in Figs. 1.h and 1.i of the manuscript. These values are from the work Takeuchi and Inoue [26], and are enthalpies calculated using Miedema's model, suitable for determining the heat formation of transition metal alloys [27]. When the mixing enthalpy of both elements is negative, the formation of precipitates is preferred. However, when it is positive, a phase separation of both elements is favored.

These enthalpies are plotted as the function of the interaction energy between a doping element X and a grain boundary ($E^{\text{inter}}_{\text{X}-\text{GB}}$) in α-iron to determine if a solute would segregate into GB. The interaction energy is calculated using:

$$E^{\text{inter}}_{\text{X}-\text{GB}} = (E^{\text{SC}}_{\text{X}-\text{GB}} - E^{\text{SC}}_{\text{GB}}) - (E^{\text{SC}}_{\text{X}-\text{bulk}} - E^{\text{SC}}_{\text{bulk}}), \tag{7}$$

These energies are provided in supplementary tables 6 and 7.

Supplementary Table 6 **Interaction energy $E^{\mathrm{inter}}_{\mathrm{X-GB}}$ for various grain boundaries determined using *ab initio* calculations.**

X	Reference	GB type	$E^{\mathrm{inter}}_{\mathrm{X-GB}}$ (eV)
H	[28]	$\Sigma5(210)$	-0.46
H	[20]	$\Sigma3(111)[110]$	-0.27
H	[21]	$\Sigma3(111)[1-10]$	-0.47
H	[24]	$\Sigma3(111)[0-11]$	-0.52
H	[25]	$\Sigma5(310)$	-0.47
H	[22]	$\Sigma5(310)$	-0.24
H	[29]	$\Sigma5(210)$	-0.81
H	[29]	$\Sigma5(310)$	-0.43
H	[29]	$\Sigma3(111)$	-0.49
H	[30]	$\Sigma5(310)$	-0.43
H	[30]	$\Sigma5(210)$	-0.13
H	[30]	$\Sigma3(111)$	-0.49
H	[31]	$\Sigma5(310)$	-0.23
B	[28]	$\Sigma5(210)$	-2.45
B	[20]	$\Sigma5(310)$	-2.07
B	[21]	$\Sigma3(111)[1-10]$	-1.96
B	[22]	$\Sigma5(310)$	-1.44
B	[32]	$\Sigma3(110)[1-10]$	-1.80
B	[33]	$\Sigma5(310)$	-2.79
B	[34]	$\Sigma3(111)$	-2.00
C	[28]	$\Sigma5(210)$	-1.80
C	[20]	$\Sigma3(111)[110]$	-0.91
C	[21]	$\Sigma3(111)[1-10]$	-1.04
C	[22]	$\Sigma5(310)$	-0.72
C	[29]	$\Sigma5(210)$	-1.58
C	[29]	$\Sigma5(310)$	-1.27
C	[29]	$\Sigma3(111)$	-0.82
C	[31]	$\Sigma5(310)$	-0.76
C	[33]	$\Sigma5(310)$	-1.76
C	[34]	$\Sigma3(111)$	-1.00
C	[35]	$\Sigma5(112)$	-0.67
C	[35]	$\Sigma5(310)$	-1.62
C	[35]	$\Sigma5(210)$	-1.77
N	Present Work	$\Sigma5(210)$	-0.77
N	Present Work	$\Sigma5(210)$	-0.74
N	[23]	$\Sigma5(210)$	-0.93
Al	[20]	$\Sigma3(111)[110]$	-0.58
Al	[21]	$\Sigma3(111)[1-10]$	-0.54
Al	[36]	$\Sigma3(111)[1-10]$	-0.50
Al	[37]	$\Sigma5(310)$	-0.41
Si	[20]	$\Sigma3(111)[110]$	-0.64
Si	[21]	$\Sigma3(111)[1-10]$	-0.63
Si	[36]	$\Sigma3(111)[1-10]$	-0.6
Si	[38]	$\Sigma3(111)$	-0.28
Si	[38]	$\Sigma3(332)$	-0.67
P	[20]	$\Sigma3(111)[110]$	-1.21
P	[24]	$\Sigma3(111)[0-11]$	-0.16
P	[29]	$\Sigma5(210)$	-0.43
P	[29]	$\Sigma5(310)$	-0.24
P	[29]	$\Sigma3(111)$	-1.14
P	[34]	$\Sigma3(111)$	-1.40
P	[36]	$\Sigma3(111)[1-10]$	-1.20
Ti	[20]	$\Sigma3(111)[110]$	-0.47
Ti	[21]	$\Sigma3(111)[1-10]$	-0.52
Ti	[32]	$\Sigma3(110)[1-10]$	-0.51
Ti	[39]	$\Sigma3(1-11)[110]$	-0.48
Ti	[39]	$\Sigma3(1-12)[110]$	-0.09
Ti	[39]	$\Sigma9(1-11)[110]$	-0.74
Ti	[39]	$\Sigma11(3-31)[110]$	-0.63
Ti	[40]	$\Sigma11(332)$	-0.50
Ti	[40]	$\Sigma3(111)[110]$	-0.37

Supplementary Table 7 **Supplementary table 6 continued.**

X	Reference	GB type	$E^{\text{inter}}_{\text{X}-\text{GB}}$ (eV)
V	[20]	$\Sigma3(111)[110]$	-0.13
V	[21]	$\Sigma3(111)[1-10]$	-0.17
V	[25]	$\Sigma5(310)$	-0.16
V	[39]	$\Sigma3(1-11)[110]$	-0.10
V	[39]	$\Sigma3(1-12)[110]$	-0.12
V	[39]	$\Sigma9(1-11)[110]$	0.01
V	[39]	$\Sigma11(3-31)[110]$	-0.20
V	[40]	$\Sigma11(332)$	-0.02
V	[40]	$\Sigma3(111)[110]$	0.01
V	[41]	$\Sigma3(111)[110]$	-0.12
Cr	[20]	$\Sigma3(111)[110]$	-0.17
Cr	[21]	$\Sigma3(111)[1-10]$	-0.14
Cr	[25]	$\Sigma5(310)$	-0.27
Cr	[39]	$\Sigma3(1-11)[110]$	-0.18
Cr	[39]	$\Sigma3(1-12)[110]$	-0.07
Cr	[39]	$\Sigma9(1-11)[110]$	0.21
Cr	[39]	$\Sigma11(3-31)[110]$	-0.29
Cr	[40]	$\Sigma11(332)$	-0.10
Cr	[40]	$\Sigma3(111)[110]$	0.00
Cr	[41]	$\Sigma3(111)[110]$	-0.12
Mn	[20]	$\Sigma3(111)[110]$	-0.46
Mn	[21]	$\Sigma3(111)[1-10]$	-0.44
Mn	[25]	$\Sigma5(310)$	-0.55
Mn	[39]	$\Sigma3(1-11)[110]$	-0.50
Mn	[39]	$\Sigma3(1-12)[110]$	-0.18
Mn	[39]	$\Sigma9(1-11)[110]$	0.54
Mn	[39]	$\Sigma11(3-31)[110]$	-0.62
Mn	[40]	$\Sigma11(332)$	-0.42
Mn	[40]	$\Sigma3(111)[110]$	-0.32
Co	[20]	$\Sigma3(111)[110]$	-0.19
Co	[39]	$\Sigma3(1-11)[110]$	-0.18
Co	[39]	$\Sigma3(1-12)[110]$	-0.04
Co	[39]	$\Sigma9(1-11)[110]$	0.14
Co	[39]	$\Sigma11(3-31)[110]$	-0.15
Co	[40]	$\Sigma11(332)$	-0.10
Co	[40]	$\Sigma3(111)[110]$	-0.10
Zr	[21]	$\Sigma3(111)[1-10]$	-1.30
Ni	[20]	$\Sigma3(111)[110]$	-0.31
Ni	[39]	$\Sigma3(1-11)[110]$	-0.42
Ni	[39]	$\Sigma3(1-12)[110]$	-0.15
Ni	[39]	$\Sigma9(1-11)[110]$	0.43
Ni	[39]	$\Sigma11(3-31)[110]$	-0.57
Ni	[40]	$\Sigma11(332)$	-0.70
Ni	[40]	$\Sigma3(111)[110]$	-0.45
Cu	[20]	$\Sigma3(111)[110]$	-0.55
Cu	[39]	$\Sigma3(1-11)[110]$	-0.51
Cu	[39]	$\Sigma3(1-12)[110]$	-0.24
Cu	[39]	$\Sigma9(1-11)[110]$	0.64
Cu	[39]	$\Sigma11(3-31)[110]$	-0.75
Cu	[40]	$\Sigma11(332)$	-0.65
Cu	[40]	$\Sigma3(111)[110]$	-0.45
Nb	[20]	$\Sigma3(111)[110]$	-0.69
Nb	[21]	$\Sigma3(111)[1-10]$	-0.97
Nb	[39]	$\Sigma3(1-11)[110]$	-0.77
Nb	[39]	$\Sigma3(1-12)[110]$	-0.16
Nb	[39]	$\Sigma9(1-11)[110]$	0.10
Nb	[39]	$\Sigma11(3-31)[110]$	-0.91
Mo	[20]	$\Sigma3(111)[110]$	-0.41
Mo	[21]	$\Sigma3(111)[1-10]$	-0.44
Mo	[41]	$\Sigma3(111)[110]$	-0.62
Mo	[39]	$\Sigma3(1-11)[110]$	-0.44
Mo	[39]	$\Sigma3(1-12)[110]$	-0.10
Mo	[39]	$\Sigma9(1-11)[110]$	0.43
Mo	[39]	$\Sigma11(3-31)[110]$	-0.54
Ta	[21]	$\Sigma3(111)[1-10]$	-0.75
W	[20]	$\Sigma3(111)[110]$	-0.37
W	[21]	$\Sigma3(111)[1-10]$	-0.41
W	[39]	$\Sigma3(1-11)[110]$	-0.40
W	[39]	$\Sigma3(1-12)[110]$	-0.08
W	[39]	$\Sigma9(1-11)[110]$	0.31
W	[39]	$\Sigma11(3-31)[110]$	-0.51
Re	[21]	$\Sigma3(111)[1-10]$	-0.28

2 Supplementary Note 2: Impact of boron addition and low-temperature tempering on reducing hydrogen embrittlement of low-carbon martensitic steel

The electron backscattered diffraction (EBSD) analysis of Fig. 2 of the manuscript shows a similar length distribution of LC, LC+B, LC+LTT, and LC+B+LTT. The microstructure's grain size (assuming a circular shape of the grain) is presented in supplementary figure 1 and shows that it is between 10 μm and 18 μm for all conditions. This result indicates that adding boron and the LTT has a minor impact on the grain size, in agreement with previous work [7, 28, 42].

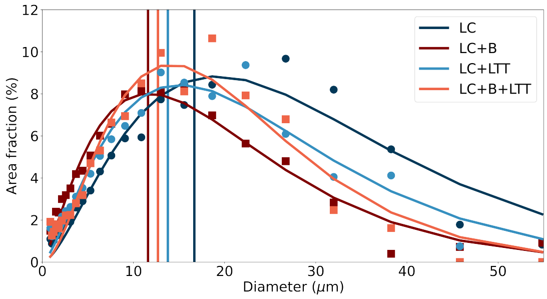

Supplementary Figure 1 Complementary information regarding the EBSD analysis of the four different microstructures investigated in this work. Grain size distribution of low-carbon steels with and without B (LC and LC+B), and with and without a low-temperature tempering (LC+LTT and LC+B+LTT).

Additional synchrotron X-ray diffraction (SXRD) measurements have been performed on systems subjected to LTT for different tempering times (t_{LTT}). Supplementary figure 2.a shows the circular integration for all microstructures, which is similar for all conditions. Supplementary figure 2.b focuses on the segment, which is mostly influenced by carbides (when 2θ is around 4.5° and 7.5°). This supplementary figure shows that without tempering, the formation of carbides is observed for both LC and LC+B, formed during the He quenching, after the martensite transformation (d auto-tempering), and has been described previously without the LTT [28]. When the tempering time increases, the signal is similar at this range, indicating a limited growth of these carbides and/or nucleation of new transition carbides. However, when t_{LTT} is increased, the austenite fraction determined by peak integration is reduced from 2.2 % to 1.6 % according to supplementary figure 2.c, with a reduction more pronounced during the first four hours. This reduction indicates that the LTT treatment induces a stress relaxation of the martensite. Additionally, the tempering induces a peak shift of both phases, which is a consequence of a change in the evolution of the lattice strain. Supplementary figures 2.d and 2.e represent the lattice parameter evolution of martensite and austenite, respectively, induced by the peak shift [43]. Since the nominal concentration of carbon is low (0.15 wt.%), the tetragonality of the martensite is less than 1% [44] and we neglect this effect to estimate the lattice parameter of the martensite. For both phases, the lattice parameter is slightly reduced when the tempering time increases, suggesting an evolution of the lattice strain. A reduction of 0.017% for martensite and 0.087% for the austenite is determined and observed during the first tempering hour. Supplementary figure 2.f plots the normalized second derivative of the austenite fraction f_g, and the lattice parameter reduction. It is observed that during the first hour of tempering, the microstructure evolution of LC and LC+B is mostly due to carbon segregation, but when the tempering time is increased, the stress relaxation is mostly due to the reduction of f_g. Hence, we observed a combined effect of both phenomena after 4h of tempering. Finally, the dislocation density for the different systems has been determined with the Williamson-Hall approach [28, 43, 45]. Supplementary figure 2.g shows that the dislocation density in both the martensite and austenite phases is almost identical ($\rho^{\alpha'}$ =1.2×10^{15} m^{-2} and ρ^{γ} = 3.8×10^{15} m^{-2}) for all t$_{LTT}$ for all conditions It indicates that the recrystallisation is minor in both phases when the tempering temperature is 160°C.

The segregation of carbon at martensite boundaries from LTT has been quantified through correlative TKD-APT measurements in Fig. 2 of the manuscript. Further, supplementary figure 3 presents the segregation of boron obtained in LC and LC+B without any LTT [28]. The TKD

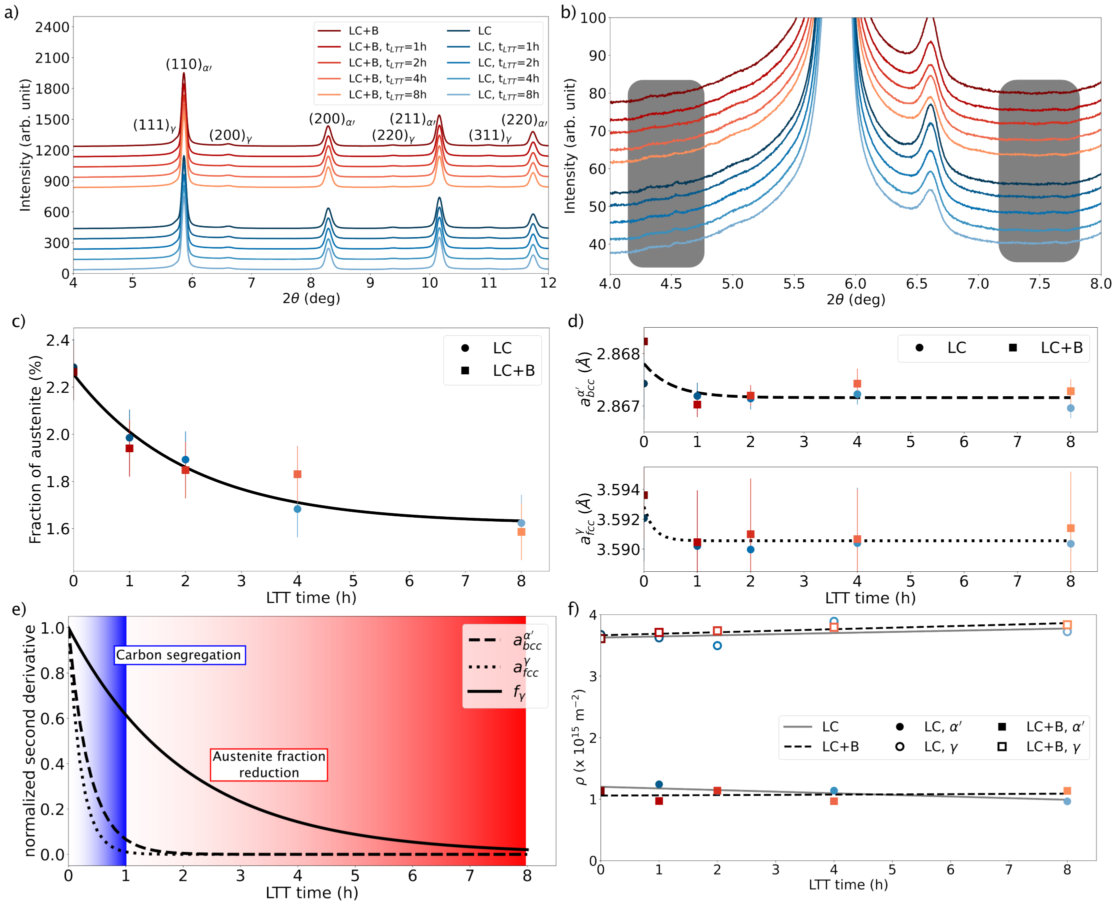

Supplementary Figure 2 Complementary information regarding the SXRD analysis of this work. a) Circular integration of the 2-dimensional diffractograms of the synchrotron X-ray measurements for LC and B+LC with different low-temperature tempering. b) Zoom of the integration to show the contribution of carbides highlighted by the grey rectangle. c) Fraction of austenite deduced from peaks integration and d) dislocation density in each phase for different times of LTT.

observation from supplementary figure 3.a shows that the misorientation angle/axis of LC and LC+B are 44°/[0.526 0.316 0.786] and 44°/[-0.743 0.656 -0.131], respectively. They are high-angle grain boundaries that do not follow the Kurdjumov-Sachs orientation relationship and can be defined as PAGBs. While the segregation of boron is barely visible in LC, a pronounced boron segregation is obtained in LC+B (supplementary figure 3.b), with an excess solute concentration of $\Gamma_B = 3.27$ at.%.nm^{-2}, according to supplementary figure 3.c.

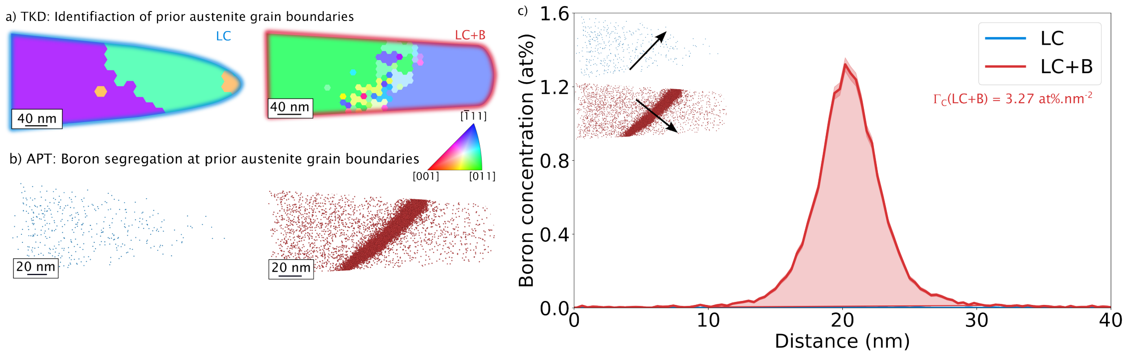

Supplementary Figure 3 Complementary information regarding the boron segregation at PAGBs in LC and LC+B [28].

3 Supplementary Note 3: Resistance against hydrogen embrittlement of the different microstructures

Fig. 3.a of the manuscript presents the residual ductility evolution when both boron addition and low-temperature tempering are applied to martensitic steels. Supplementary figures 4.a and 4.b of this supplementary information presents the tensile test curves for each condition used to determine the residual ductility. Fig. 3 .a of the manuscript highlights a significant behavior difference between the tempered and untempered conditions for both systems with and without boron.

Additional experiments have been performed at higher current density to increase the hydrogen activity. Supplementary figure 4.c presents the hydrogen concentration after 1 h of charging for LC, LC+B, LC+LTT, and LC+B+LTT. While the concentration of hydrogen is below 5 wppm for LC+LTT when the current density varies from -1.25 mA.cm^{-2} to -6.25 mA.cm^{-2}, all others conditions have an increase of C_H. It suggests the impact of the solute segregation becomes limited for more aggressive conditions, but still shows a better resistance against hydrogen embrittlement, as shown in supplementary figure 4.d. A slightly better ductility is observed for LC+B+LTT than LC, LC+B, and LC+LTT, even if the ductility is below 5 %. In the case of LC+LTT, even if the total hydrogen concentration should be lower than 5 wppm, a brittle fracture is observed, most likely because prior austenite boundaries are not protected by boron, which induces a premature failure of the system in a hydrogen environment.

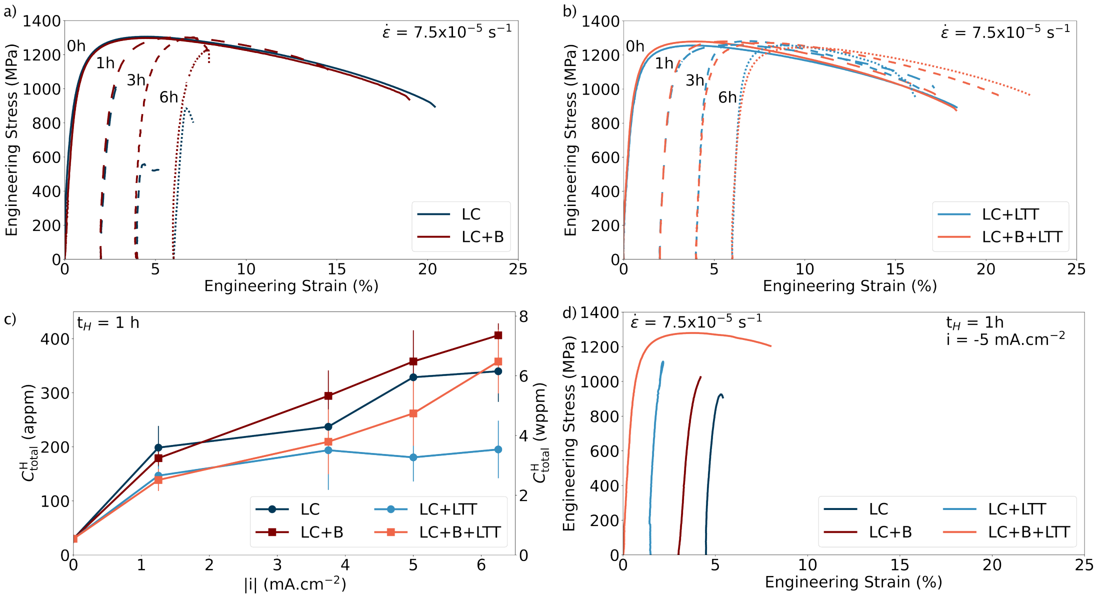

Supplementary Figure 4 Complementary information regarding the improved resistance against hydrogen embrittlement of tempered and un-tempered boron-doped and boron-free steels. Engineering stress-strain curves of H-precharged a) LC, LC+B, b) LC+LTT and LC+B+LTT. c) hydrogen concentration evolution with increased current density for 1 h charging, and d) Tensile stress-strain of H-precharged LC, LC+B, LC+LTT, and LC+B+LTT with a current density of -5 mA.cm^{-2}

Fig. 3.b of the manuscript also shows a pronounced reduction in hydrogen ingress when LC and LC+B are tempered. Such results have been obtained because of the stress relaxation and carbon segregation at defects where hydrogen could segregate (*e.g.*: martensite boundaries) during the tempering. Further investigation has been conducted to estimate the evolution of the hydrogen diffusion coefficient on tempered and untempered steels. For this study, we use an electrolytic cell with two compartments (one for the cathodic polarization and the other for the anodic polarization), which are both equipped with reference electrodes Ag/AgSO$_4$ and platinum counter electrodes [46]. The cathodic cell was an aqueous solution with 0.5 M NaOH, and the anodic cell was also an aqueous solution but containing only 0.2 M of NaOH. Between both solutions, the working electrode is the studied sample. For the permeation tests, samples were austenitized in a furnace for

110 s, then water-quenched to obtain a similar grain size as the one prepared in a Bähr dilatometer. Supplementary figure 5.a presents the permeation curves obtained for both LC and LC+LTT. The permeation test shows that the steady state current density is slightly lower for LC+LTT than for LC, consistent with the lower solubility of hydrogen observed using the TDS when steel is tempered. Additionally, the apparent hydrogen diffusion coefficient D_{app} of both LC and LC+LTT can be estimated through the permeation experiments, by fitting the following equation [47]:

$$\frac{d\ln j}{dt} = -\frac{1}{2t} + \frac{L^2}{D_{app}t^2} \tag{8}$$

with t the permeation time and L the thickness of the specimen.

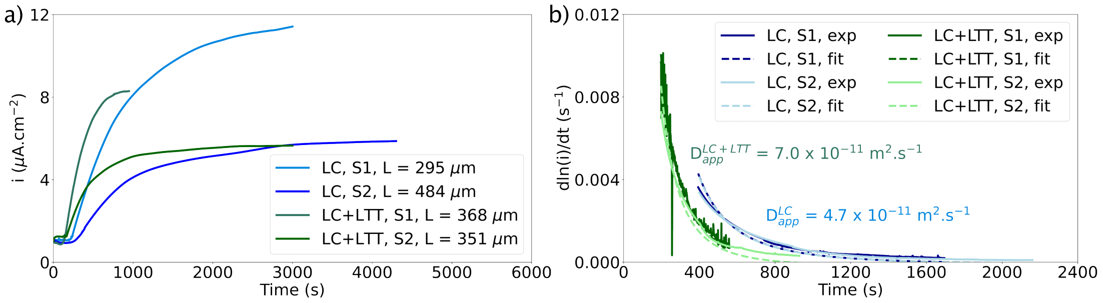

Supplementary Figure 5 Permeation tests performed on LC and LC+LTT. a) Current density i as a function of time and b) their corresponding derivative.

From supplementary figure 5.b, the apparent hydrogen diffusion coefficient of LC is higher than for LC+LTT ($D_{app}^{\mathrm{LC}} = 4.7 \pm 1.8 \times 10^{-11}$ m^2.s^{-1} and $D_{app}^{\mathrm{LC+LTT}} = 7.0 \pm 0.2 \times 10^{-11}$ m^2.s^{-1}). This result is consistent with our observations: low-temperature tempering increases the apparent hydrogen diffusion coefficient due to fewer trapping sites from carbon segregation.

4 Supplementary Note 4: Secondary crack analysis of hydrogen pre-charged tensile test specimens

Supplementary figures 6, 7, 8, and 9 present the secondary cracks used to quantify the distribution SCs in Fig. 4.e of the manuscript, which are observed in a hydrogen pre-charged (t_h = 6 h) and tensile-strained LC, LC+B, LC+LTT, and LC+B+LTT, respectively. For all images, only cracks along PAGBs, MBs, inclusion interfaces, {001}, and {011} planes are considered and plotted in different colors.

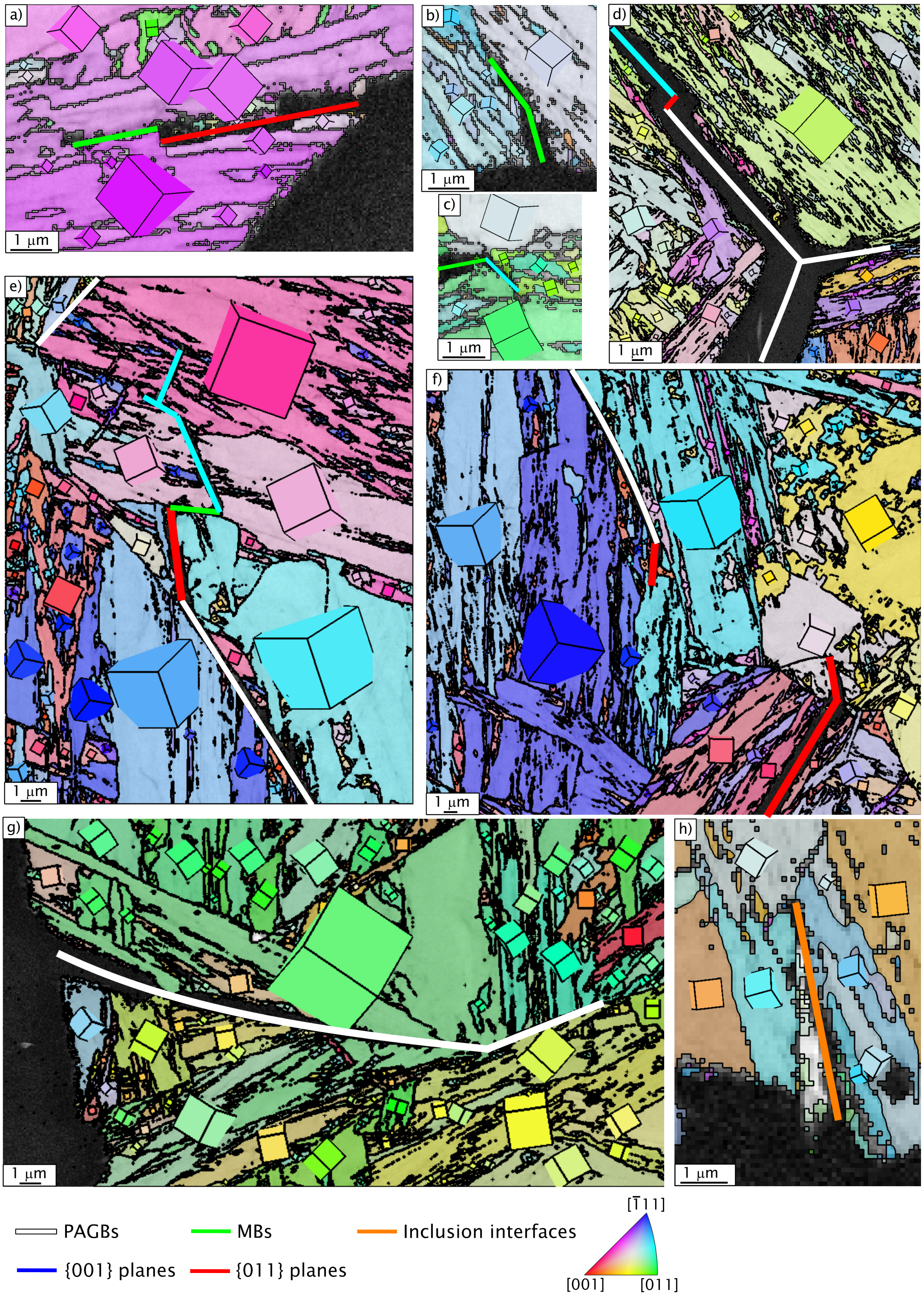

Supplementary Figure 6 Secondary cracks observed in hydrogen pre-charged LC.

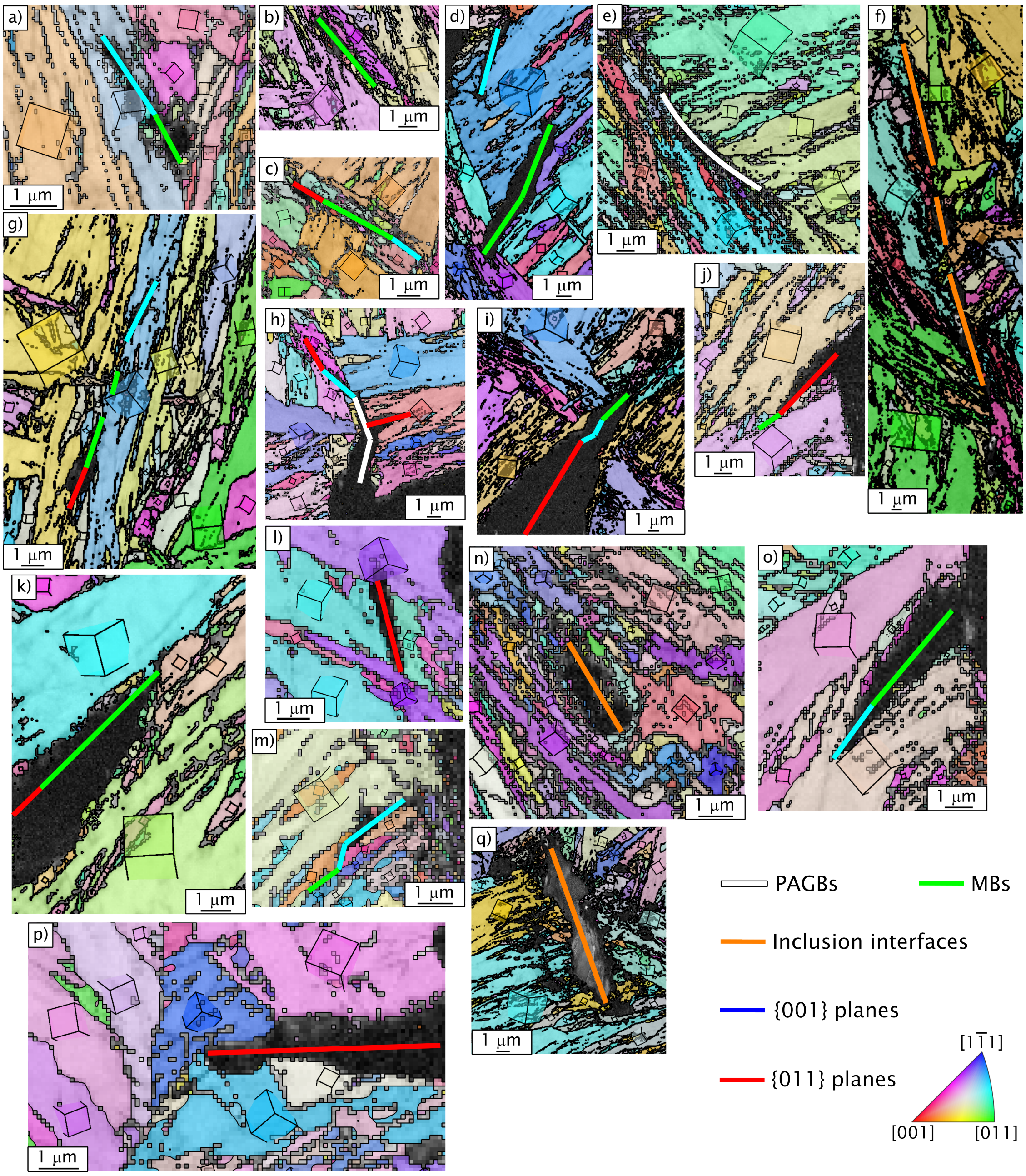

Supplementary Figure 7 Secondary cracks observed in hydrogen pre-charged LC+B.

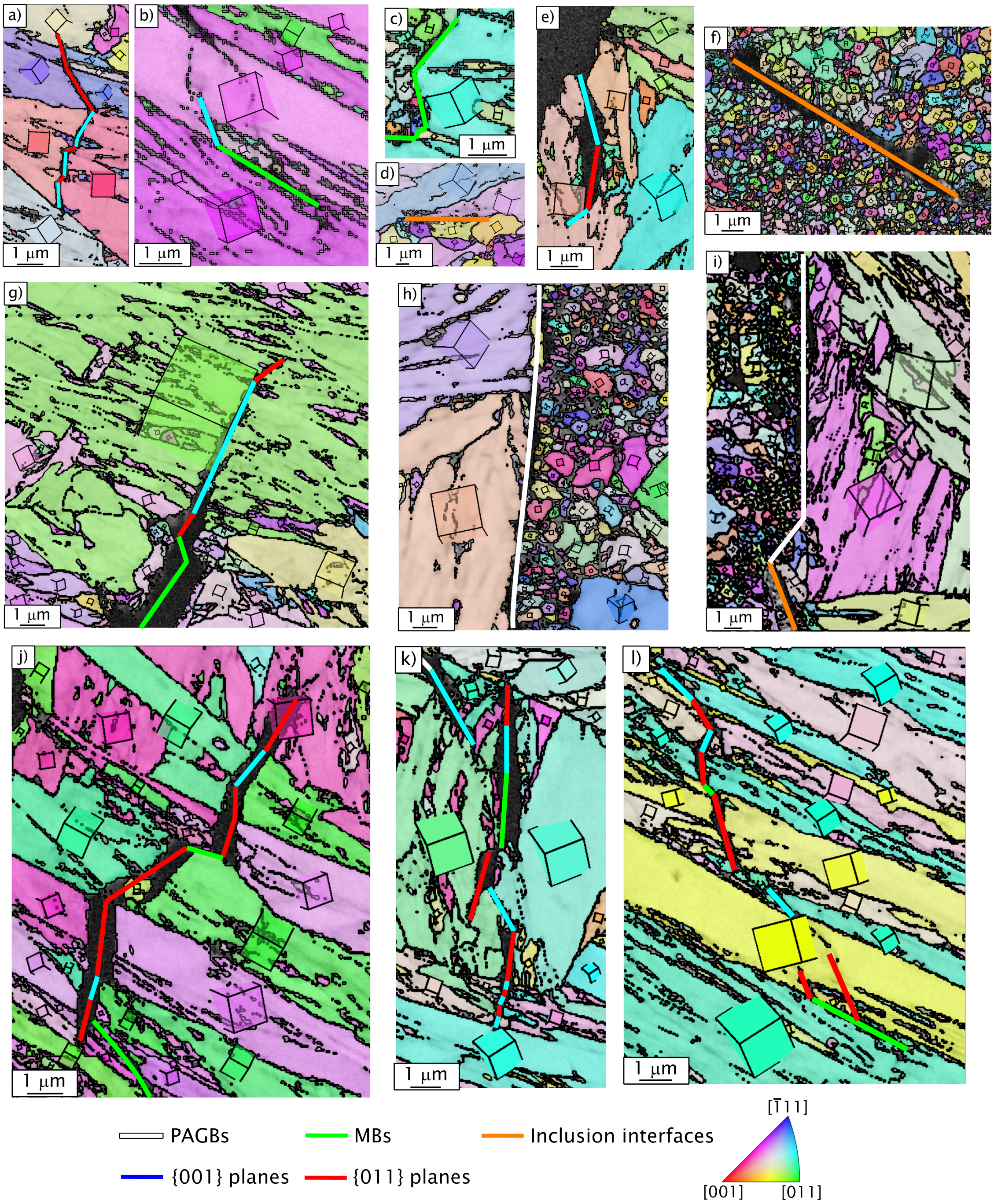

Supplementary Figure 8 **Secondary cracks observed in hydrogen pre-charged LC+LTT.**

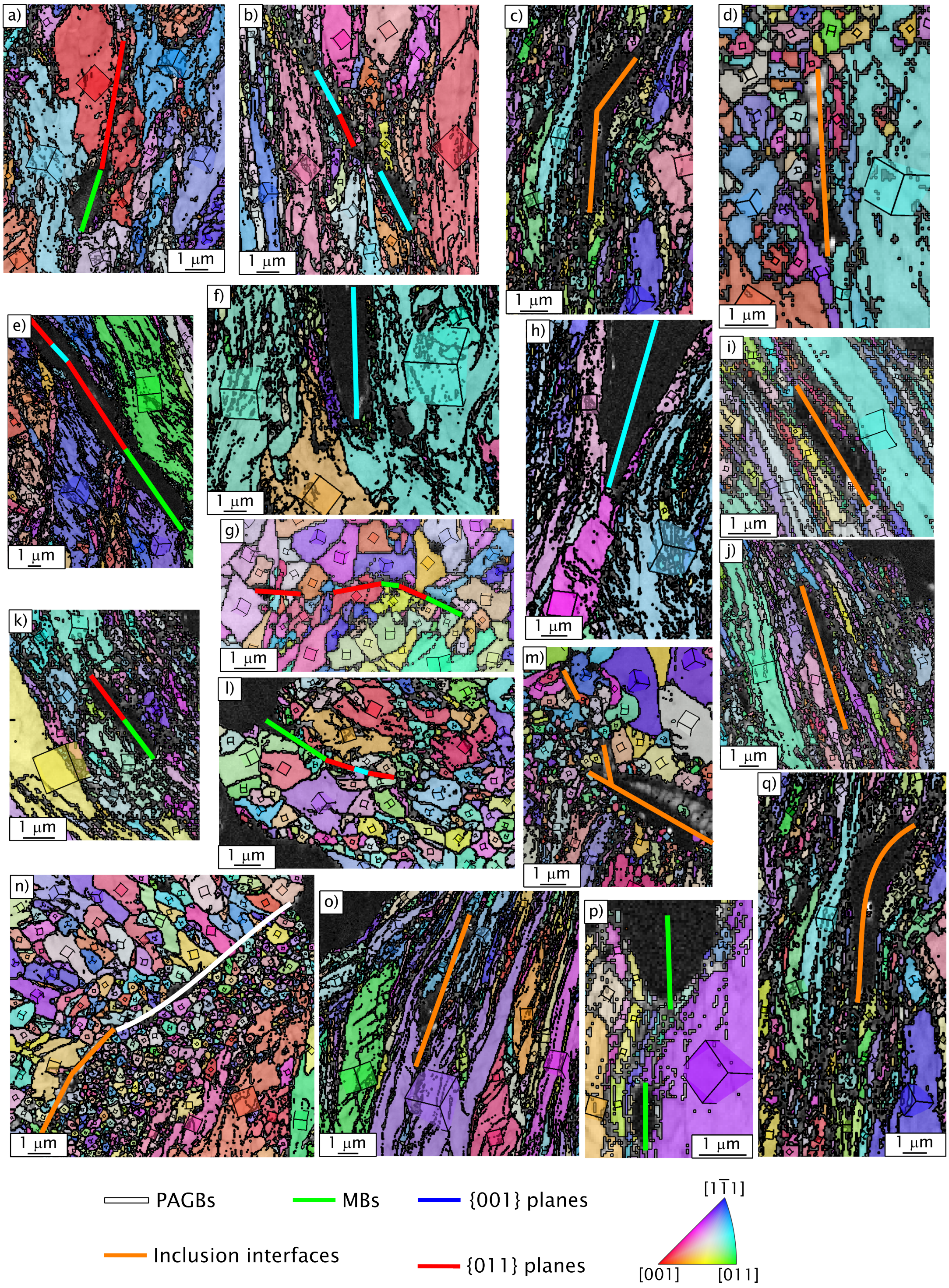

Supplementary Figure 9 **Secondary cracks observed in hydrogen pre-charged LC+B+LTT.**

5 Supplementary Note 5: Retained austenite and residual stress analysis

The concentration of hydrogen near the α'/γ interface (shown in Fig. 5.c of the manuscript) highlights an increase in the austenite. Since the sample was not pre-charged electrochemically, this hydrogen concentration may be either residual hydrogen, introduced during sample preparation, or derived from gas ionization during APT analysis. Hydrogen detected through gas ionization is directly linked to the variation in field evaporation, a topic extensively discussed previously [48]. Such a phenomenon can be made apparent by tracking the relative ratio between the different charge states of elements [49]. Supplementary figure 10.b plots the concentration of Fe^{+}, and Fe^{2+}, which are directly dependent on the electrostatic field. While the concentration of Fe^{2+} fluctuates depending on the concentration of manganese, it can be seen that the concentration of Fe^{+} decreases in γ, indicating a higher field in γ than in α'. Since a higher field should result in less hydrogen originating from the residual gas [48], the detected background hydrogen in γ should be lower than in α'. Consequently, the hydrogen concentration measured in γ should mostly be from solute hydrogen incorporated either from sample preparation or residual in the system. In addition, Fig. 5.c of the manuscript shows that almost no carbon partitioning is observed in the retained austenite, even though a tempering of 4h at 160° has been performed on the system. In this particular case, this absence of carbon partitioning is noted because the interface is next to a grain boundary, where carbon is primarily segregating, as plotted in supplementary figure 10.d.

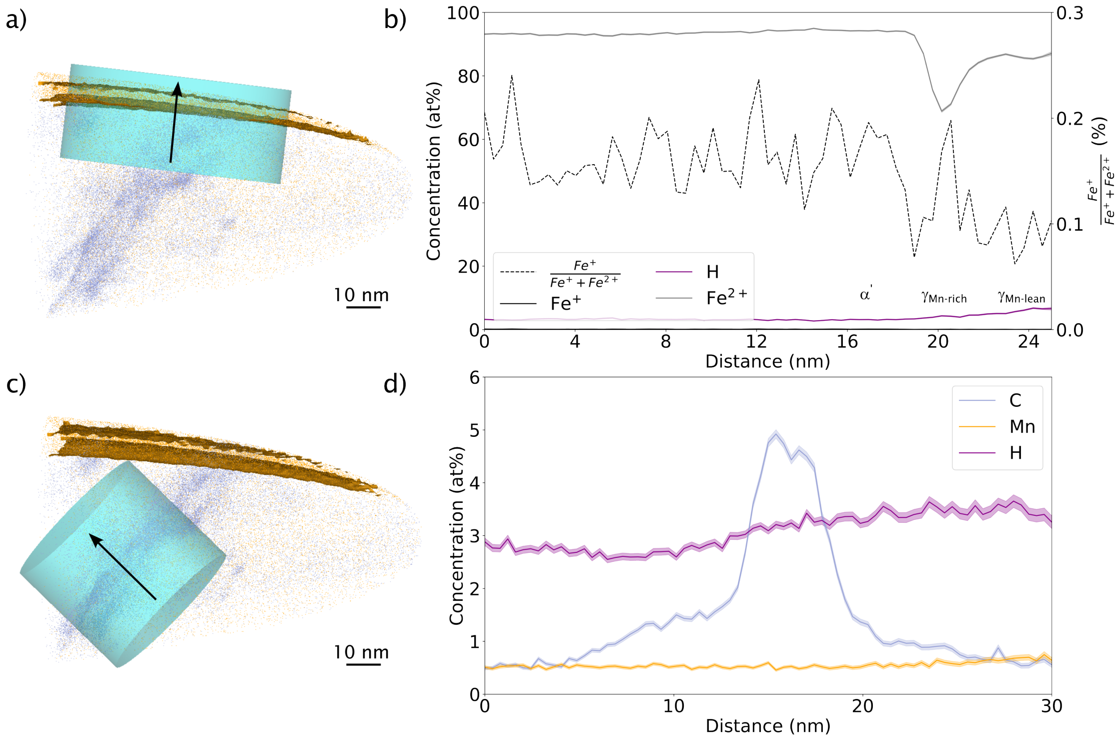

Supplementary Figure 10 Complementary information on the retained austenite detected through APT. a) Reconstruction focusing on the α'/γ interface and b) relative composition of Fe^{+} and Fe^{2+} measured across the interface. c) Reconstruction focusing on the grain boundaries next to the interface with the corresponding relative composition of C, Mn, and H.

Finally, additional SXRD measurements have been performed on tensile-strained LC, LC+B, LC+LTT, and LC+B+LTT with and without hydrogen pre-charged for 3 h. These experiments aimed to estimate the austenite fraction depending on the local strain with and without hydrogen. Hence, different measurements have been performed at 1 mm, 2 mm, and 4 mm from the fracture surface, which corresponds to different local strains measured using the DIC systems before the specimen fracture (supplementary figure 11.a).

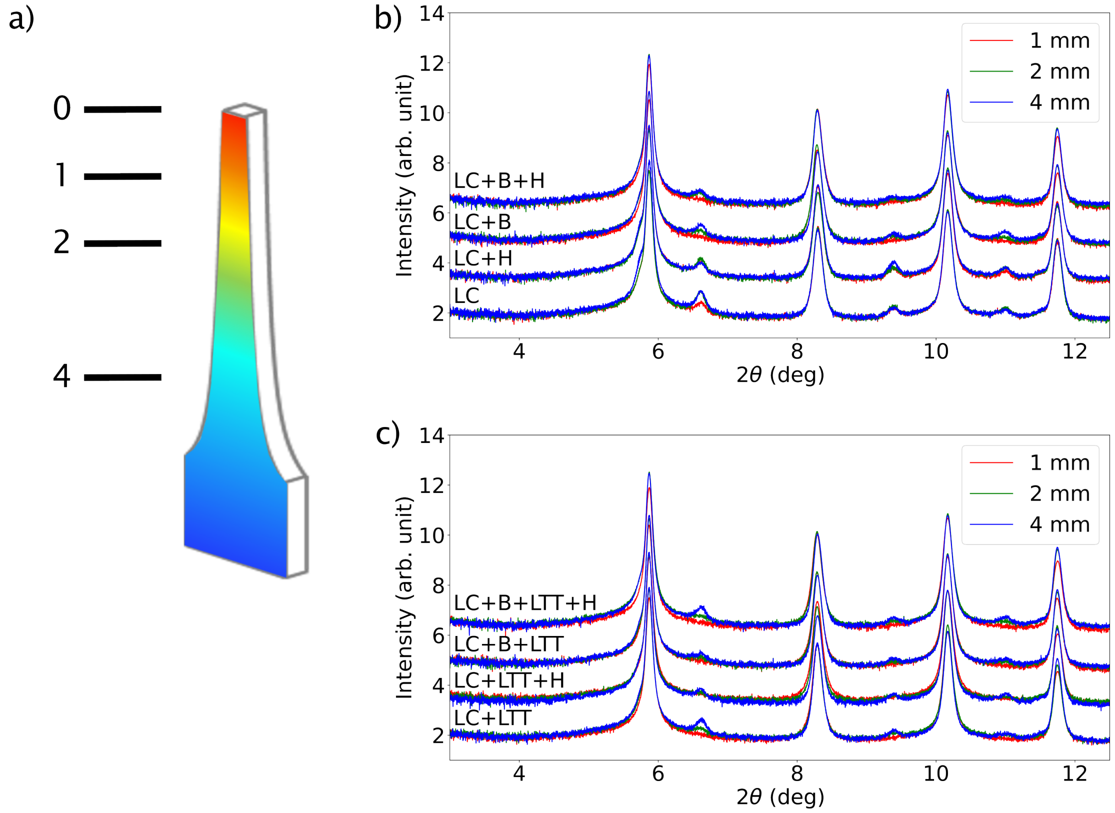

Supplementary Figure 11 Complementary information regarding the synchrotron X-Ray diffraction measurements presented in Fig. 5 of the manuscript. a) The incident synchrotron beam went at 1 mm, 2 mm and 4 mm from the fracture surface. Corresponding circular integration along the axis direction for steel b) without and c) with the LTT applied.

Supplementary figures. 11.b and 11.c present the circular integration along the axis direction for the different specimens. For all integration, peaks related to austenite are reduced when the measurement is closer to the fractured surface (larger local strain), indicating a deformation-induced martensite transformation (DIMT) effect. However, when boron is not added or tempering is conducted, a large fraction of retained austenite is still observed, indicating that the DIMT effect is minor. Such a phenomenon is expected when the plastic strain is localized at prior austenite boundaries, which are not strengthened with boron or additional carbon from LTT (in the case of LC and LC+H).

Since no early deformation-induced martensite transformation (DIMT) is seen for hydrogen-precharged tempered steel, this austenite is either more stable due to stress relaxation or/and the hydrogen uptake in this phase is reduced from carbon partitioning in this phase. From synchrotron experiments, the measured lattice parameter change from tempering (Δ_{LTT}) was -0.087 % for the austenite and -0.017 % for the martensite. The equivalent hydrostatic stress $\sigma_{\mathrm{hydro}}^{\alpha',\gamma}$ from the reduced lattice parameter can be estimated using [50]:

$$\sigma_{\mathrm{hydro}}^{\alpha',\gamma} = \frac{E^{\alpha',\gamma}}{1-2\nu}\Delta_{\mathrm{LTT}}^{\alpha',\gamma}. \tag{9}$$

with $E^{\alpha',\gamma}$ the isotropic Young's modulus of martensite and austenite ($E^{\alpha'}$ = 180 GPa and E^{γ} = 172 GPa) and ν the Poisson's ratio (ν = 0.3) [50]. It leads to an increase in the compression stress of 374 MPa for the austenite and a reduction of the tensile stress of 76 MPa for the martensite when the system is tempered. Considering that the fraction of austenite is 2.2 % in these steel grades, the global hydrostatic stress relaxation from tempering $\sigma_{\mathrm{hydro}}^{\mathrm{SXRD}}$ = -86 MPa.

The residual stress reduction from tempering has also been measured using FIB-DIC ring-core for LC and LC+LTT because it allows the measurement of the actual residual stress based on the

measurement of relaxation strains caused by material removal at the micro scale [51, 52]. They have been carried out using ThermoFisher Helios 5UX DualBeam with a current of 440 pA and an acceleration voltage of 30 kV for the incremental annular. SEM imaging was acquired using 0.8 nA current and 5 kV acceleration voltage between every milling step. The resulting deformations on the surface were measured by digital image correlation (DIC) using commercial software VIC-2D. In this work, two pillars have been milled for both LC and LC+LTT samples. The pillar, wall, and speckle surface diameters are 7 μm, 5 μm, and 3 μm, respectively Supplementary figures 12.a and 12.b present the measured $\Delta\varepsilon_{xx}$, $\Delta\varepsilon_{yy}$, and $\Delta\varepsilon_{xy}$ that are the image horizontal, vertical directions, and shear strains, respectively, during milling each pillar.

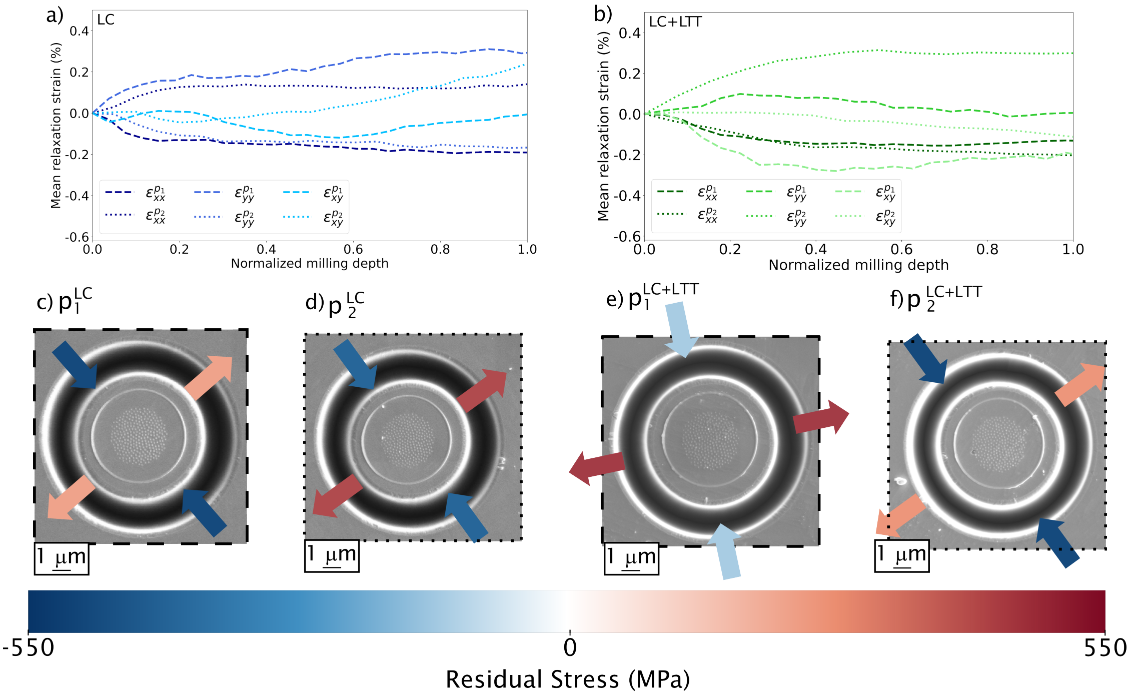

Supplementary Figure 12 Complementary experiments to evaluate the stress relaxation from tempering on LC and LC+LTT. Horizontal $\Delta\varepsilon_{xx}$, $\Delta\varepsilon_{yy}$ vertical, and shear $\Delta\varepsilon_{xy}$ strains measured by DIC during milling micro-pillar on a) LC and b) LC+LTT. Milled pillars in c,d) LC and e,f) LC+LTT with the deduced stress in directions 1 and 2.

Then, the strain tensor is expressed in the principal base as follows:

$$\Delta\varepsilon = \begin{bmatrix} \Delta\varepsilon_1 & 0 \\ 0 & \Delta\varepsilon_2 \end{bmatrix} \tag{10}$$

using plane strain Mohr's circle derivation:

$$\Delta\varepsilon_{1|2} = \frac{\Delta\varepsilon_{xx} + \Delta\varepsilon_{yy}}{2} \pm \sqrt{\left(\frac{\Delta\varepsilon_{xx} - \Delta\varepsilon_{yy}}{2}\right)^2 + \Delta\varepsilon_{xy}^2} \tag{11}$$

And

$$\theta = \tan^{-1}\left(\frac{2\Delta\varepsilon_{xy}}{\Delta\varepsilon_{xx} - \Delta\varepsilon_{yy}}\right) \tag{12}$$

Directions 1 and 2 are offset by angle θ from the image base. The transformation into the principal base allows the calculation of the residual stress state using homogeneous isotropic Hooke's Law:

$$\begin{bmatrix} \sigma_1 \\ \sigma_2 \end{bmatrix} = -\frac{E}{(1-\nu)^2} \begin{bmatrix} 1 & \nu \\ \nu & 1 \end{bmatrix} \begin{pmatrix} \Delta\varepsilon_1^\infty \\ \Delta\varepsilon_2^\infty \end{pmatrix} \tag{13}$$

where $\Delta\varepsilon_{1|2}^\infty$ the saturated relaxation strain. This saturation is reached when no further relaxation strains are observable upon milling. It has been shown that it occurs when the milling depth

is equivalent to the pillar diameter with finite element modelling (*i.e.*: when the normalized milling depth is 1) [53]. Therefore, $\sigma_{1|2}$ are determined using strain at this depth level. For every pillar, the stress state in the principal base is represented in supplementary figures 12.c-f and the estimated values are reported in supplementary table 8.

Supplementary Table 8 Measured internal stress released during the ring core experiments.

	p_1^{LC}	p_2^{LC}	p_1^{LC+LTT}	p_2^{LC+LTT}
σ_1	-487 MPa	-405 MPa	-127 MPa	-520 MPa
σ_2	223 MPa	432 MPa	471 MPa	256
θ	9°	36°	12°	36°

It is observed that high residual stresses are measured from both systems with and without tempering, suggesting a limited effect of the heat treatments. We also noted that the fluctuation between each pillar is non-negligible and mostly due to the lath martensite microstructure, which is heterogeneous [54]. However, an average hydrostatic stress ($\sigma_{\text{hydro}} = 1/2(\sigma_1 + \sigma_2)$) can still be estimated for LC and LC+LTT which are $\sigma_{\text{hydro}}^{LC}$ = -60 MPa and $\sigma_{\text{hydro}}^{\text{LC+LTT}}$ = 20 MPa. These values indicate a difference of $\sigma_{\text{hydro}}^{\text{Ring-Core}}$ of 80 MPa, similar to $\sigma_{\text{hydro}}^{\text{SXRD}}$ estimated previously. It, hence, supports the measured $\sigma_{\text{hydro}}^{\alpha'}$ = -76 MPa and $\sigma_{\text{hydro}}^{\gamma}$ = -374 MPa observed after 4 h of tempering at 160°C.

References

[1] Zhang, S. et al. Mitigation of hydrogen embrittlement in ultra-high strength lath martensitic steel via Ta microalloying. Materials and Design **210**, 110090 (2021).

[2] Kim, H. et al. Effects of titanium content on hydrogen embrittlement susceptibility of hot-stamped boron steels. Journal of Alloys and Compounds **735**, 2067–2080 (2018).

[3] Momotani, Y., Shibata, A., Terada, D. & Tsuji, N. Effect of strain rate on hydrogen embrittlement in low-carbon martensitic steel. International Journal of Hydrogen Energy **42**, 3371–3379 (2017).

[4] Okada, K. et al. Improvement of resistance against hydrogen embrittlement by controlling carbon segregation at prior austenite grain boundary in 3Mn-0.2C martensitic steels. Scripta Materialia **224**, 115043 (2023).

[5] Yoo, J. et al. Effects of solid solution and grain-boundary segregation of Mo on hydrogen embrittlement in 32MnB5 hot-stamping steels. Acta Materialia **207**, 116661 (2021).

[6] Shibata, A. et al. Microstructural and crystallographic features of hydrogen-related fracture in lath martensitic steels. Materials Science and Technology **33**, 1524–1532 (2017).

[7] Shi, H. et al. Improving hydrogen embrittlement resistance of martensitic steel via interface boron segregation. Tech. Rep., Max Planck Institute for Sustainable Materials (2024).

[8] Han, J., Nam, J. & Lee, Y. The mechanism of hydrogen embrittlement in intercritically annealed medium Mn TRIP steel. Acta Materialia **113**, 1–10 (2016).

[9] Sun, B. et al. Chemical heterogeneity enhances hydrogen resistance in high-strength steels. Nature Materials **20**, 1629–1634 (2021).

[10] Shen, X. et al. Influence of microstructural morphology on hydrogen embrittlement in a medium-Mn steel Fe-12Mn-3Al-0.05C. Metals **9** (2019).

[11] Jeong, I. et al. Austenite morphology and resistance to hydrogen embrittlement in medium Mn transformation-induced plasticity steel. Scripta Materialia **169**, 52–56 (2019).

[12] Sun, J., Jiang, T., Sun, Y., Wang, Y. & Liu, Y. A lamellar structured ultrafine grain ferrite-martensite dual-phase steel and its resistance to hydrogen embrittlement. Journal of Alloys and Compounds **698**, 390–399 (2017).

[13] Dong, F. et al. The influence of phosphorus on the temper embrittlement and hydrogen embrittlement of some dual-phase steels. Materials Science and Engineering: A **854**, 143379 (2022).

[14] Drexler, A. et al. Influence of Plastic Deformation on the Hydrogen Embrittlement Susceptibility of Dual Phase Steels. Key Engineering Materials **926 KEM**, 2077–2091 (2022).

[15] Wang, Z., Liu, J., Huang, F., Bi, Y. & Zhang, S. Hydrogen Diffusion and Its Effect on Hydrogen Embrittlement in DP Steels With Different Martensite Content. Frontiers in Materials **7**, 1–12 (2020).

[16] Iacoviello, F., Habashi, M. & Cavallini, M. Hydrogen embrittlement in the duplex stainless steel Z2CND2205 hydrogen-charged at 200Â°C. Materials Science and Engineering: A **224**, 116–124 (1997).

[17] Arniella, V., Álvarez, G., Belzunce, J. & Rodríguez, C. Hydrogen embrittlement of 2205 duplex stainless steel in in-situ tensile tests. Theoretical and Applied Fracture Mechanics **124** (2023).

[18] Vaňová, P. & Sojka, J. Hydrogen embrittlement of duplex steel tested using slow strain rate test. Metalurgija **53**, 163–166 (2014).

[19] Zakroczymski, T., Glowacka, A. & Swiatnicki, W. Effect of hydrogen concentration on the embrittlement of a duplex stainless steel. Corrosion Science **47**, 1403–1414 (2005).

[20] Song, K., Cao, S., Bao, Y., Qian, P. & Su, Y. Designing hydrogen embrittlement-resistant grain boundary in steel by alloying elements segregation: First-principles calculations. Applied Surface Science **656**, 159684 (2024).

[21] Kholtobina, A., Ecker, W., Pippan, R. & Razumovskiy, V. Effect of alloying elements on hydrogen enhanced decohesion in bcc iron. Computational Materials Science **188**, 110215 (2021).

[22] Kulkov, S. S., Bakulin, A. V. & Kulkova, S. E. Effect of boron on the hydrogen-induced grain boundary embrittlement in α-Fe. International Journal of Hydrogen Energy **43**, 1909–1925 (2018).

[23] Matsumoto, R., Riku, M., Taketomi, S. & Miyazaki, N. Hydrogen-Grain Boundary Interaction in Fe, Fe-C, and Fe-N Systems. Progress in Nuclear Science and Technology **2**, 9–15 (2011).

[24] Schuler, T., Christien, F., Ganster, P. & Wolski, K. Ab initio investigation of phosphorus and hydrogen co-segregation and embrittlement in α-Fe twin boundaries. Applied Surface Science **492**, 919–935 (2019).

[25] Subramanyam, A., Guzmán, A., Vincent, S., Hartmaier, A. & Janisch, R. Ab initio study of the combined effects of alloying elements and h on grain boundary cohesion in ferritic steels. Metals **9** (2019).

[26] Takeuchi, A. & Inoue, A. Classification of bulk metallic glasses by atomic size difference, heat of mixing and period of constituent elements and its application to characterization of the main alloying element. Materials Transactions **46**, 2817–2829 (2005).

[27] Miedema, A. R. Simple Model for Alloys I. Rules for the alloying behaviour of transition metals. Philips Technical Review **33**, 149–160 (1973).

[28] Hachet, G. et al. Segregation at prior austenite grain boundaries: the competition between boron and hydrogen. International Journal of Hydrogen Energy **95**, 734–746 (2024).

[29] Verkhovykh, Đ., Mirzoev, Đ. & Dyuryagina, N. Ab Initio Modeling of Interactions of P, H, C, S With Grain Boundaries in α-Iron. Bulletin of the South Ural State University series: Mathematics. Mechanics. Physics **13**, 57–68 (2021).

[30] Mirzaev, D., Mirzoev, A., Okishev, K. & Verkhovykh, A. Ab initio modelling of the interaction of H interstitials with grain boundaries in bcc Fe. Molecular Physics **114**, 1502–1512 (2016).

[31] Tahir, A. M., Janisch, R. & Hartmaier, A. Hydrogen embrittlement of a carbon segregated σ5 (310) [001] symmetrical tilt grain boundary in α-Fe. Materials Science and Engineering: A **612**, 462–467 (2014).

[32] Sawada, H., Haga, J. & Ushioda, K. First-principles Calculation of Interaction between Boron Atom and Transition Metal Elements in α-Fe: Effect of Boron on Recrystallization Behavior in Ti Added Ultra-low Carbon Cold-rolled Steel Sheets. Tech. Rep. 120, Advanced Technology Research Laboratories (2018).

[33] Wang, J., Enomoto, M. & Shang, C. First-principles study on the P-induced embrittlement and de-embrittling effect of B and C in ferritic steels. Acta Materialia **219**, 117260 (2021).

[34] Yamaguchi, M. First-principles study on the grain boundary embrittlement of metals by solute segregation: Part I. iron (Fe)-solute (B, C, P, and S) systems. Metallurgical and Materials Transactions A **42**, 319–329 (2011).

[35] Wang, J., Janisch, R., Madsen, G. K. & Drautz, R. First-principles study of carbon segregation in bcc iron symmetrical tilt grain boundaries. Acta Materialia **115**, 259–268 (2016).

[36] Ito, K., Sawada, H., Tanaka, S., Ogata, S. & Kohyama, M. Electronic origin of grain boundary segregation of Al, Si, P, and S in bcc-Fe: Combined analysis of ab initio local energy and crystal orbital Hamilton population. Modelling and Simulation in Materials Science and Engineering **29** (2021).

[37] Řehák, P., Všianská, M. & Černý, M. Role of vibrational entropy in impurity segregation at grain boundaries in bcc iron. Computational Materials Science **216**, 111858 (2023).

[38] Bhattacharya, S., Kohyama, M., Tanaka, S. & Shiihara, Y. Si segregation at Fe grain boundaries analyzed by ab initio local energy and local stress. Journal of Physics Condensed Matter **26** (2014).

[39] Mai, H., Cui, X., Scheiber, D., Romaner, L. & Ringer, S. The segregation of transition metals to iron grain boundaries and their effects on cohesion. Acta Materialia **231**, 117902 (2022).

[40] Xu, Z., Tanaka, S. & Kohyama, M. Grain-boundary segregation of 3d-transition metal solutes in bcc Fe: Ab initio local-energy and d-electron behavior analysis. Journal of Physics Condensed Matter **31** (2019).

[41] Tian, Z. X., Yan, J. X., Hao, W. & Xiao, W. Effect of alloying additions on the hydrogen-induced grain boundary embrittlement in iron. Journal of Physics Condensed Matter **23** (2011).

[42] Sharma, M., Ortlepp, I. & Bleck, W. Boron in Heat-Treatable Steels: A Review. Steel Res. Int. **90**, 1–28 (2019).

[43] Wei, S., Kang, J. & Tasan, C. An in situ synchrotron x-ray study of reverse austenitic transformation in a metastable femnco alloy. Journal of Materials Research **38**, 281–296 (2023).

[44] Tanaka, T., Maruyama, N., Nakamura, N. & Wilkinson, A. Tetragonality of fe-c martensite - a pattern matching electron backscatter diffraction analysis compared to x-ray diffraction. Acta Materialia **195**, 728–738 (2020).

[45] Williamson, G. K. & Smallman, R. E. The use of Fourier analysis in the interpretation of X-ray line broadening from cold-worked iron and molybdenum. Acta Crystallographica **7**, 574–581 (1954).

[46] Lu, X., Wang, D. & Johnsen, R. Hydrogen diffusion and trapping in nickel-based alloy 625: An electrochemical permeation study. Electrochimica Acta **421** (2022).

[47] Frappart, S. et al. Study of the hydrogen diffusion and segregation into fecmo martensitic hsla steel using electrochemical permeation test. Journal of Physics and Chemistry of Solids **71**, 1467–1479 (2010).

[48] Chang, Y. et al. Characterizing solute hydrogen and hydrides in pure and alloyed titanium at the atomic scale. Acta Materialia **150**, 273–280 (2018).

[49] Kingham, D. The post-ionization of field evaporated ions: A theoretical explanation of multiple charge states. Surface Science **116**, 273–301 (1982).

[50] Gong, W. et al. Lattice parameters of austenite and martensite during transformation for fe-18ni alloy investigated through in-situ neutron diffraction. Acta Materialia **250**, 118860 (2023).

[51] Lunt, A. & Korsunsky, A. A review of micro-scale focused ion beam milling and digital image correlation analysis for residual stress evaluation and error estimation. Surface and Coatings Technology **283**, 373–388 (2015).

[52] Zhao, Z. D. et al. Mitigating hydrogen embrittlement in a 1.8 gpa-grade press-hardened steel by internal stress relaxation via low-temperature tempering. Scripta Materialia **266** (2025).

[53] Korsunsky, A., Sebastiani, M. & Bemporad, E. Residual stress evaluation at the micrometer scale: Analysis of thin coatings by fib milling and digital image correlation. Surface and Coatings Technology **205**, 2393–2403 (2010).

[54] Archie, F., Mughal, M., Sebastiani, M., Bemporad, E. & Zaefferer, S. Anisotropic distribution of the micro residual stresses in lath martensite revealed by fib ring-core milling technique. Acta Materialia **150**, 327–338 (2018).
